# An essential role of *Plasmodium berghei *kinesin 8 in axoneme assembly and male gametogenesis

**DOI:** 10.1186/2046-2530-4-S1-P46

**Published:** 2015-07-13

**Authors:** D Depoix, S Marques, D Ferguson, T Duguet, T Vucanovic, R Sinden, P Grellier, L Kohl

**Affiliations:** 1 UMR7245, MNHN, Paris, France; 2 Department of Life Sciences, Imperial College London, London, UK; 3 Nuffield Department of Clinical Laboratory Science, University of Oxford, Oxford, UK

## Objective

The male gamete of the *Plasmodium *parasite is the only developmental stage that possesses a flagellum. Very little is known about the identity and function of the proteins involved in the parasite's flagellum assembly. Assembly is intracytoplasmic, IFT independent and extremely fast. To understand this essential step of the parasite life cycle, we focused on a male gametocyte and gamete specific kinesin (Kin8), initially identified by proteomic analysis.

## Methods

Kinesin 8 knock-out parasites were constructed, cloned and their ability to form male gametes *in vitro*, to fertilize female *in vivo *and to pursue their life cycle was assessed. The ultrastructure of the male gametocytes/gametes was studied in detail.

## Results

Kin8 mutant lines produce male and female gametocytes similar to WT parasites but male gametogenesis was severely impaired as male gametocytes were not able to release male gametes (no exflagellation). The ultrastucture analysis revealed a default in axoneme assembly: elongated microtubules were seen in longitudinal sections but the classical 9+2 axoneme structure was never observed. Nevertheless, Kin8 KO mutants were able to form some ookinetes *in vivo *and could be transmitted by the mosquito to a new host.

## Conclusion

We characterized a kinesin essential for male gametogenesis and axoneme assembly in *Plasmodium berghei*, providing new insights into *Plasmodium *flagellar organization.

Further characterization and the protein localisation are ongoing.

